# Thermometric lateral flow immunoassay with colored latex beads as reporters for COVID-19 testing

**DOI:** 10.1038/s41598-022-07963-1

**Published:** 2022-03-10

**Authors:** Terumitsu Azuma, Yuen Yung Hui, Oliver Y. Chen, Yuh-Lin Wang, Huan-Cheng Chang

**Affiliations:** 1 Institute of Atomic and Molecular Sciences, Academia Sinica, Taipei, 106 Taiwan, ROC; 2 Department of Physics, National Taiwan University, Taipei, 106 Taiwan, ROC; 3 Department of Chemical Engineering, National Taiwan University of Science and Technology, Taipei, 106 Taiwan, ROC; 4 Department of Chemistry, National Taiwan Normal University, Taipei, 106 Taiwan, ROC

**Keywords:** Biotechnology, Chemistry, Materials science

## Abstract

Temperature sensing is a promising method of enhancing the detection sensitivity of lateral flow immunoassay (LFIA) for point-of-care testing. A temperature increase of more than 100 °C can be readily achieved by photoexcitation of reporters like gold nanoparticles (GNPs) or colored latex beads (CLBs) on LFIA strips with a laser power below 100 mW. Despite its promise, processes involved in the photothermal detection have not yet been well-characterized. Here, we provide a fundamental understanding of this thermometric assay using non-fluorescent CLBs as the reporters deposited on nitrocellulose membrane. From a measurement for the dependence of temperature rises on the number density of membrane-bound CLBs, we found a 1.3-fold (and 3.2-fold) enhancement of the light absorption by red (and black) latex beads at 520 nm. The enhancement was attributed to the multiple scattering of light in this highly porous medium, a mechanism that could make a significant impact on the sensitivity improvement of LFIA. The limit of detection was measured to be 1 × 10^5^ particles/mm^2^. In line with previous studies using GNPs as the reporters, the CLB-based thermometric assay provides a 10× higher sensitivity than color visualization. We demonstrated a practical use of this thermometric immunoassay with rapid antigen tests for COVID-19.

## Introduction

The COVID-19 epidemic has emerged as a major public health concern since its first identification in Wuhan, China, on December 2019. Over the past 2 years, tremendous efforts have been made on a global level to fight the SARS-CoV-2 virus^1^. Alongside disease research and vaccine development, immunodiagnostics has been extensively carried out worldwide to understand and decipher the disease states of patients. Although various types of detection tools have been available^2^, there is still an urgent need for rapid, quantitative, and sensitive immunodiagnostic platforms in the combat against COVID-19^3^. These technologies are fundamental to the development of better methods to prevent and control the epidemic, whose spread is exponentially growing and intrinsically unpredictable^4^.

Enzyme-linked immunosorbent assay (ELISA) and lateral flow immunoassay (LFIA) are two commonly used immunodiagnostic tools^5^. Both methods are based on specific antigen–antibody reactions. While ELISA has been serving as the gold standard in the field for decades, the assay is time-consuming and costly. Particularly, it requires secondary antibodies linked with enzymes (such as horseradish peroxidase and alkaline phosphatase) as reporters in the assays and also needs significant expertise to carry out the tests. LFIA, in contrast, is fast, low-cost, and scalable. It can rapidly diagnose a sample containing antigens or antibodies of interest by capillary flow across a membrane strip in typically 15 min^6,7^. LFIA first appeared in commercial use as a general-purpose pregnancy test in 1988 and has become one of the most promising methods for point-of-care testing (POCT)^8^, including one-step home diagnosis of COVID-19 today.

Colloidal gold nanoparticles (GNPs) are the most widely used reporters in LFIA because the assay can be conducted colorimetrically by eye without the need of an instrument^9,10^. The technique takes advantage of the fact that GNP exhibits an exceptionally strong absorption in the optical region, known as surface plasmon resonance (SPR)^11^, caused by the interaction between light and electrons on the surface of GNPs. For spherical GNPs of 40 nm in diameter (i.e. particles typically used in LFIA)^12^, the SPR band has a molar extinction coefficient of 8.42 × 10^9^ /M/cm or an absorption cross section of about 3 × 10^–11^ cm^2^/particle at 530 nm^13,14^. The limit of detection (LOD) of these particles in the test zone of a LFIA strip has been reported to be 3.78 × 10^6^ particles/mm^2^, measured by using a photo scanner for the color intensity in the blue channel^15^. Although this color readout approach is direct and low-cost, the sensitivity is relatively low, with a LOD of 1–10 ng/mL for the analyte concentration.

In view of this deficiency, various techniques have been developed to enhance the sensitivity as well as the quantification capability of the GNP-based LFIA. These techniques include surface-enhanced Raman scattering (SERS), photothermal detection (PTD), and photoacoustic (PA) detection, as comprehensively reviewed by Ye et al.^16^. Among these methods, PTD is most practical and has readily achieved a 10× improvement in detection sensitivity^17–20^. A PTD system typically consists of a continuous-wave laser as the energy source to heat up the reporters, which absorb visible light and then emit infrared photons. An infrared camera measures the temperature rises of the reporters trapped in control and test zones of the strips. Since the amount of the heat generated is linearly proportional to the amount of the trapped particles before absorption saturation, this thermometric method is well suited for quantitative analysis.

The objective of this work is to provide a fundamental understanding of this so-called thermometric lateral flow immunoassay (TLFIA) using non-fluorescent red and black CLBs (diameter of 0.4 μm) as the reporters and a green laser (wavelength of 520 nm) as the heat source. The reasons to choose CLBs in this study are twofold. First, the applicability of PTD to CLBs in LFIA is not yet known. Second, CLBs outperform GNPs in terms of multiplexing capability because organic dyes of different colors can be uniformly incorporated into the polystyrene matrix^21^. For fluorescent polystyrene particle of 40 nm in diameter, each of them can contain more than 300 dye equivalents^22^. Gaigalas et al*.*^23^ have reported an absorption cross section of $\sigma$ ≈ 2 × 10^–8^ cm^2^/particle for 2.5-µm polystyrene spheres doped with green fluorescent dyes. If one assumes a constant number density of dye molecules in particles of different sizes, scaling this value linearly with their volume suggests a cross section of $\sigma$ ≈ 8 × 10^–11^ cm^2^/particle for 0.4-μm CLBs. This number is 3× that of 40-nm GNPs and is expected to be further increased for CLBs containing non-fluorescent dyes with a concentration of up to 20%^24^.

Here, we provide a systematic investigation on the laser-induced temperature changes as a function of the number density of CLBs on nitrocellulose (NC) membrane, which is a key component of LFIA and also a highly light scattering medium. In particular, experiments are performed with a home-built device to assess important parameters such as effective laser powers used in the excitation as well as effective absorption cross sections of the membrane-bound CLBs involved in the PTD. Understanding in depth these light absorption processes is essential to the establishment of a better and higher-sensitivity POCT platform. The utility of TLFIA is finally demonstrated with an immunoassay for nucleocapsid proteins of the SARS-CoV-2 virus.

## Results

### Turbidity

**Figure 1:**
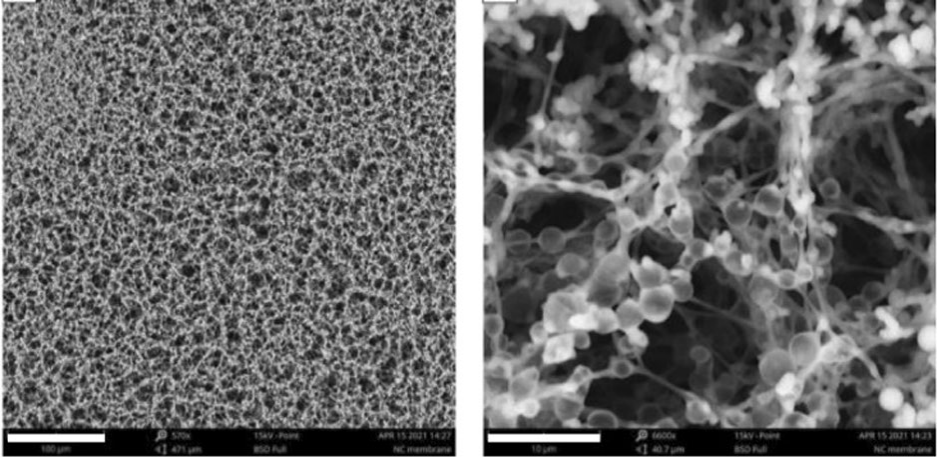
SEM images of NC membrane. SEM analysis of the structure of NC membrane with a magnification of 570× (left) and 6600× (right). Scale bars: 100 µm (left) and 10 µm (right).

Prior to the PTD experiments, knowing the structure of the NC membrane is crucial. SEM serves well the purpose. Figure 1 presents two SEM images of a typical NC membrane used in this experiment. As seen, the NC membrane has a highly fibrous and porous architecture, with a pore size of about 5–15 μm in diameter. The porosity is 70–80%^25^, and thus the membrane can scatter light strongly in the visible region. A significant intensity attenuation occurs when light passes through it. Turbidity ($\tau$) is a useful parameter to describe this phenomenon, defined as^26^

1 $$\tau =-\frac{ln\left(I/{I}_{0}\right)}{d}=-\frac{\mathit{ln}\left[({I}_{0}-{I}_{s})/{I}_{0}\right]}{d},$$

where ${I}_{0}$ is the incident light intensity, $I$ is the transmitted light intensity, ${I}_{s}$ is the intensity of light scattered by the NC membrane, and $d$ is the membrane thickness. We measured the turbidity by use of a continuous-wave 520-nm laser and found $\tau$ = 1.6 × 10^2^/cm for the dry NC membrane after properly taking into account the reflection loss of light from the polystyrene backing. The high turbidity suggests that most of the light scattered by the NC fibers in the membrane will have directions differing from that of the incoming beam. Multiple scattering occurs, resulting in a longer path of the light through the sample. In the presence of absorbing particles like CLBs in the matrix, the scattering can lead to multiple absorption of light by the same particles^27^, which in effect increases their absorption cross sections. It points out an interesting possibility of utilizing the multiscattering effect to enhance the detection sensitivity of LFIA.

### Photothermal detection

**Figure 2:**
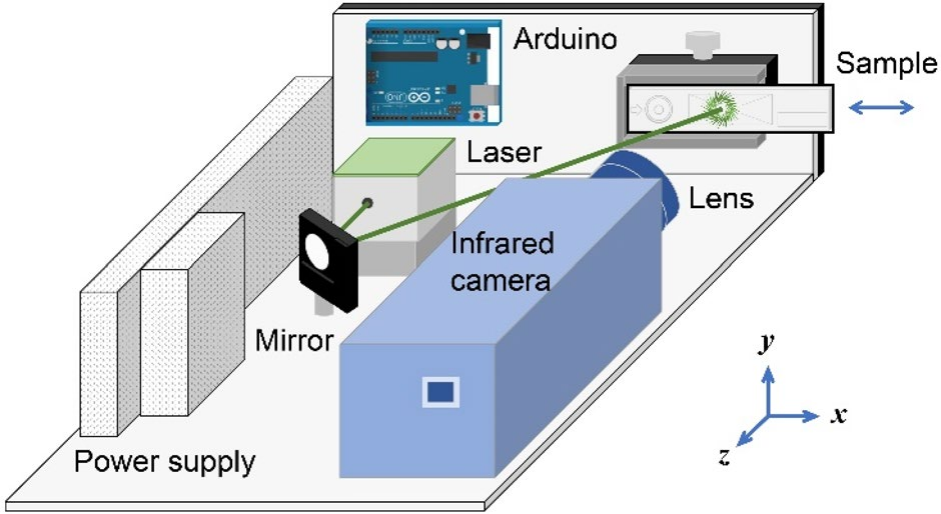
Instrument layout of the TLFIA reader. Major components of the instrument are labeled in the figure and described in text. The instrument is portable, having dimensions of 32 cm (length) × 20 cm (width) × 12 cm (height).

**Figure 3:**
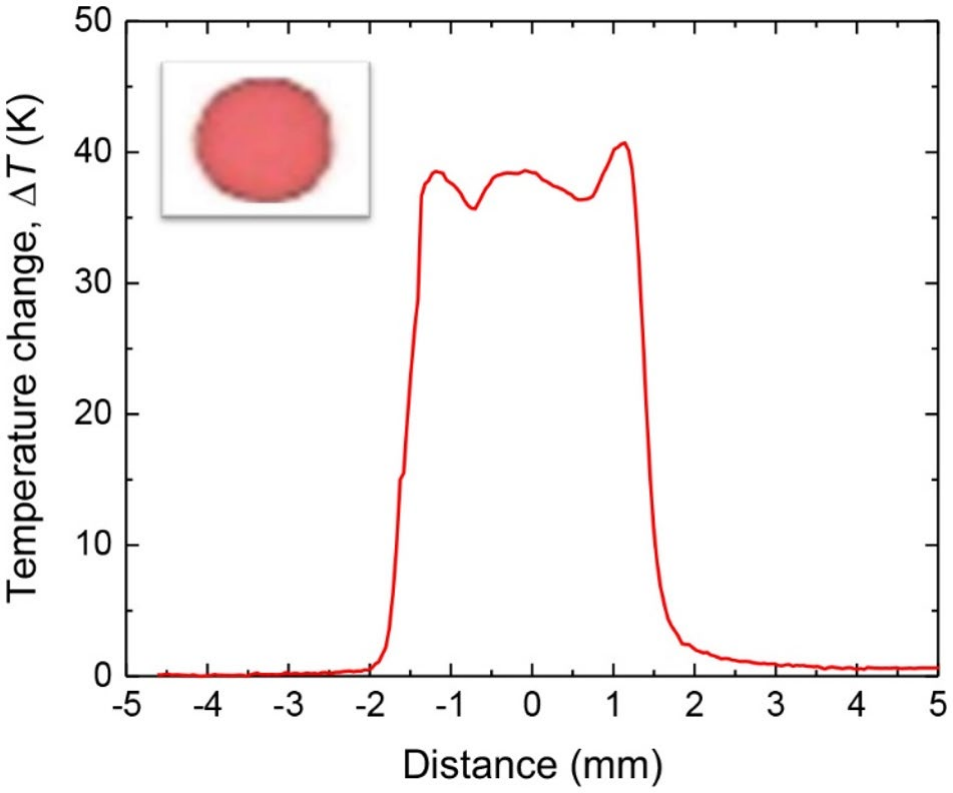
Temperature profile of a laser-irradiated CLB spot on NC membrane. The temperature is measured by using the infrared camera when the motorized translation stage moves along the *x* direction as defined in Fig. 2. The corresponding optical image of the spot made of red latex beads is shown in the inset. The number density of the particles deposited on the membrane is 7.5 × 10^11^ particles/cm^3^.

Figure 2 presents an instrument layout of a home-built TLFIA reader used in this experiment. It consists of a high-performance infrared camera and a continuous-wave laser operating at 520 nm. The laser has a specified beam diameter of 0.6 mm (1/*e*^2^), corresponding to a full width at half maximum (FWHM) of 0.35 mm. It is unfocused and irradiated onto the sample at an incident angle of ~ 45° along the long axis of the NC membrane strip, with an elliptical beam shape of 0.49 × 0.35 mm^2^ on the sample surface. This laser-irradiated area is smaller than the standard dimensions (4 × 1 mm^2^) of both control and test lines printed on LFIA strips^12^. To test the performance of the instrument for PTD of CLBs, we prepared the sample by dropping 0.5 μL of red latex bead solution (4% w/w or 1.05 × 10^12^ particles/mL) onto the NC membrane. The CLBs formed a coffee-ring-like structure with a diameter of 3.0 mm^15^, as shown in the inset of Fig. 3 for the optical image of the sample. Given this spot size and the NC membrane thickness of 0.1 mm, a mean number density of 7.5 × 10^11^ particles/cm^3^ was calculated for the CLBs distributed uniformly in the membrane.

**Figure 4:**
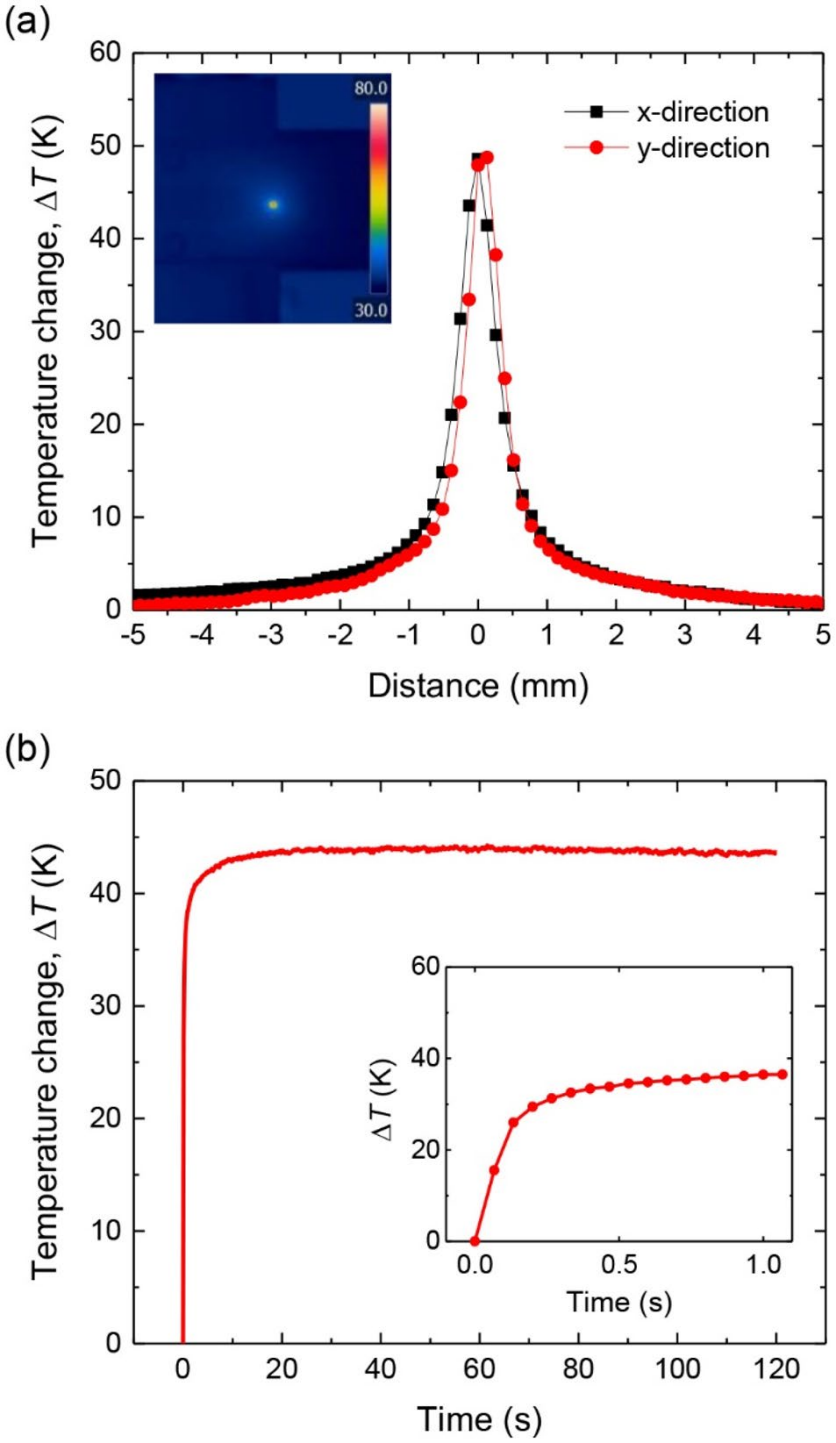
Spatial and temporal temperature profiles of laser-irradiated CLBs on NC membrane. (**a**) Temperature changes of laser-irradiated red latex beads on NC membrane in *x* and *y* directions, defined in Fig. 2. The number density of the particles deposited on the membrane is 7.5 × 10^11^ particles/cm^3^. Inset: thermal image of a laser-irradiated spot. (**b**) Time-dependent temperature changes of laser-irradiated red latex beads on NC membrane. Inset: enlarged view of the time evolutions over 0–1 s.

We next measured the temperature rises of the red spot using the TLFIA reader with the laser irradiating at its center. The power of the laser was intentionally kept low (10 mW) to avoid photodamage of the dye molecules embedded in the microspheres. The inset in Fig. 4a shows a thermal image of the sample when the laser was irradiated at the center of the red spot. The steady-state temperature profile of the image was slightly asymmetric with a FWHM of 0.77 mm along the *x* direction and 0.64 mm along the *y* direction. The result coincides with the elliptical shape of the laser beam projected on the strip surface over an area of 0.49 × 0.35 mm^2^. The significant difference between these two areas signifies the occurrence of heat dissipation over a region larger than that covered by the laser beam.

The infrared camera installed in the TLFIA reader has a response time of 12 ms^28^. Taking advantage of this feature, we explored further the temporal temperature profiles of the laser-irradiated areas. Figure 4b displays a temporal profile of the measured temperature rise as a function of the excitation time. The data presented in the profile are the maximum temperature rises measured within the laser irradiation region over time. As seen, the temperature rise reached its steady-state value of more than 40 °C in 30 s with a time constant of 0.12 s, which is not instrument-limited. It demonstrates that the radiation energy absorbed by CLBs could be rapidly transferred to the NC membrane and released as heat, similar to that of colloidal gold^17^. No noticeable photodamage of the particles was found within the temperature measurement time of 120 s. In separate experiments, we found that an increase of the local temperature in the 0.49 × 0.35 mm^2^ region above 100 °C could create a burn spot on the membrane, which occurred if a laser power of 40 mW (or a power density of 40 W/cm^2^) was applied to the same sample described above.

To further examine the performance of the TLFIA reader for real-world applications, we employed the red spot to simulate the CLBs captured on the control and test lines of a LFIA strip. Specifically, we obtained the cross-sectional temperature profile of the spot by moving the strip mounted on a motorized translation stage to allow the laser beam to come across the center of the red spot. Displayed in Fig. 3 is a typical result of the thermometric measurement for the red latex beads exposed to the 520-nm laser having an output power of 10 mW. With the stage moving at a speed of 0.6 mm/s, a temperature rise of 40 °C was readily observed at the spot center. Compared with the steady-state value measured for the same sample, this temperature rise is about 15% smaller primarily due to the reduction of the laser exposure time (cf. Fig. 4b).

### Enhanced absorption

To analyze the photothermal process, we seek to understand first how light is absorbed by CLBs in this highly scattering medium. We referred to the modified Beer–Lambert law, which was developed as a basis for near-infrared spectroscopy of biological tissues^29,30^. The non-linear terms, which appear only at high laser intensities^31^, were not included in the analysis. The law treats the illuminated tissue, which is highly scattering also, as being optically homogeneous as

2 $$-ln(I/{I}_{0})={\mu }_{a}L+G,$$

where ${\mu }_{a}$ is the absorption coefficient of tissue and $L$ is the total mean path length of detected photons, and $G$ is a geometry-dependent factor representing the intensity loss caused by light scattering. Given ${\mu }_{a}=\sigma N$ in our case, Eq. (2) becomes

3 $$-ln(I/{I}_{0})=\sigma NL+G={\sigma }_{eff}Nd+G,$$

where $N$ is the number density of absorbing particles in the membrane, and $\sigma$ and ${\sigma }_{eff}\equiv \sigma L/d$ are the absorption cross section and effective absorption cross section of the membrane-bound particles, respectively. Note that in this equation, although the membrane-bound CLBs themselves also contribute to multiscattering, the effect is small compared with that caused by the medium alone, particularly at low number density regions. To simplify subsequent analysis, we assume that the intensity loss due to the light scattering by CLBs in the NC matrix is negligible, i.e. $G=\tau d$, and write

4 $$-ln(I/{I}_{0})=({\sigma }_{eff}N+\tau )d.$$

**Figure 5:**
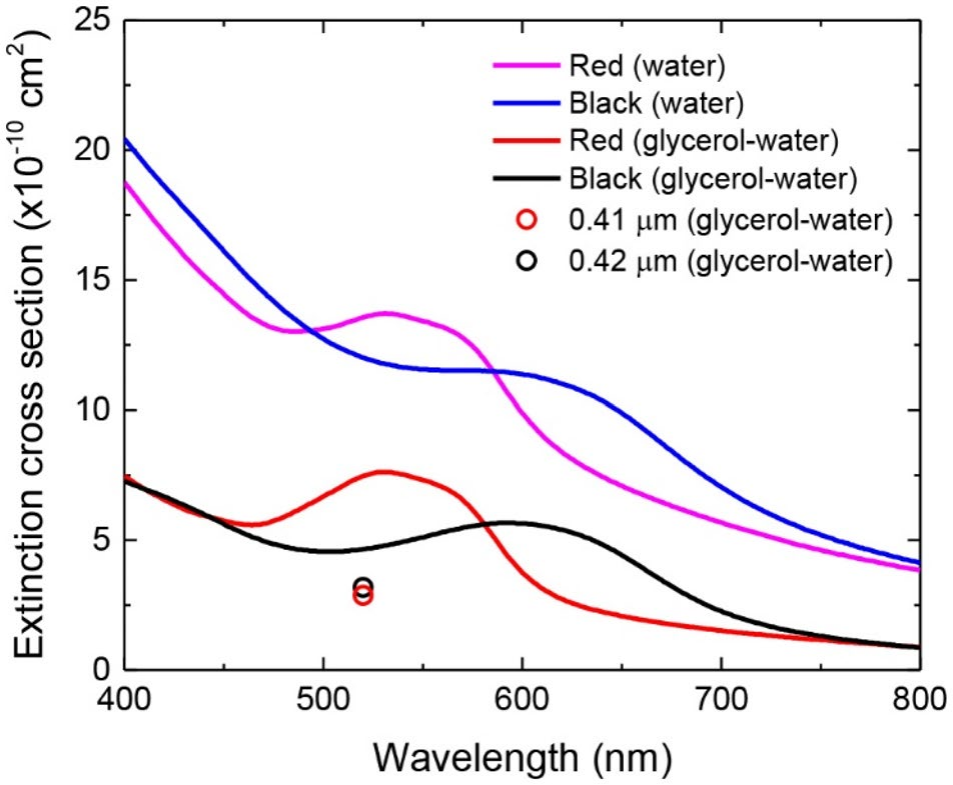
Extinction spectra of CLBs in solution. The spectra are measured for red and black latex beads suspended in water and 90% glycerol/water. Concentrations of both beads in the solutions are 0.004%. Red and black open circles are calculated scattering cross sections at 520 nm, predicted by Mie calculations for undyed polystyrene microspheres (diameters of 0.41 μm and 0.42 μm) in 90% glycerol/water.

Before our measurements for ${\sigma }_{eff}$, we first determined $\sigma$ by acquiring the extinction spectra of red and black latex beads in water after 1000× dilution of their stock solutions (4% w/w). As shown in Fig. 5, a large background associated with Mie scattering of the sub-micron particles was clearly visible in the individual spectra. Although both the beads showed significant absorption at 520 nm, their absorption cross sections could not be precisely determined due to the lack of knowledge of the corresponding scattering cross sections. To deduce such information, we performed Mie scattering calculations for undyed polystyrene microspheres in water, following the work of He et al.^32^. Using undyed polystyrene microspheres of 356 ± 14 nm and 465 ± 11 nm in diameter as the samples, the authors demonstrated that it is possible to achieve near perfect agreement between experiments and calculations for these particles dispersed in water after properly taking into account the wavelength dependence of the refractive indexes (${n}_{p}$ and ${n}_{w}$) of polystyrene and water. However, in the present study, the diameter, size deviation, and shape uniformity of the CLBs used are not exactly known. In order to minimize the errors caused by these uncertainties, we measured the absorption strengths of both CLBs dispersed in a glycerol/water mixture (glycerol:water = 9:1, v/v) to reduce the light scattering effect^33^. From the well-documented refractive indexes of water and glycerin^34^, we calculated a refractive index of ${n}_{gw}$ = 1.4607 at 520 nm for the mixture. The value can be compared with ${n}_{p}$ = 1.6000 of polystyrene at the same wavelength^34^. Although there remains a refractive index mismatch, we corrected the effect by Mie scattering calculations for undyed polystyrene microspheres of 0.41 µm and 0.42 µm in diameter in the mixture. Figure 5 displays the measured extinction scattering cross sections of both beads dispersed in 90% glycerol/water as well as their scattering-resulted extinctions calculated with the Mie theory at 520 nm. From the differences between measured and calculated values, we estimated an absorption cross section of $\sigma$ = 4.6 × 10^–10^ cm^2^/particle and $\sigma$ = 1.5 × 10^–10^ cm^2^/particle for the red and black latex beads, respectively.

For CLBs, one would expect their absorption cross sections to be unchanged after deposition on the NC membrane since the dye molecules responsible for light absorption are incorporated into the interior of the polystyrene spheres. In contrast, the SPR bands of GNPs may markedly be altered after deposition as the particles can easily form aggregates on the NC membrane (Supplementary Fig. S1). The difference renders CLBs more appealing than GNPs in the present study. Despite this advantage, issues associated with multiscattering of light within the highly porous membrane are still a concern. The multiscattering can not only give rise to an enhancement of light absorption as discussed earlier, but also result in an attenuation of the excitation light intensity. To experimentally measure ${\sigma }_{eff}$ in Eq. (4), we used the TLFIA reader to investigate how the temperature rises varied with the CLB concentrations of the droplets deposited on the strips. The PTD serves well for the purpose because it has been previously demonstrated that the photothermal spectra obtained for absorbing molecules or nanoparticles dispersed in scattering medium corresponds to the absorption component of the sample’s extinction^35–37^.

**Figure 6:**
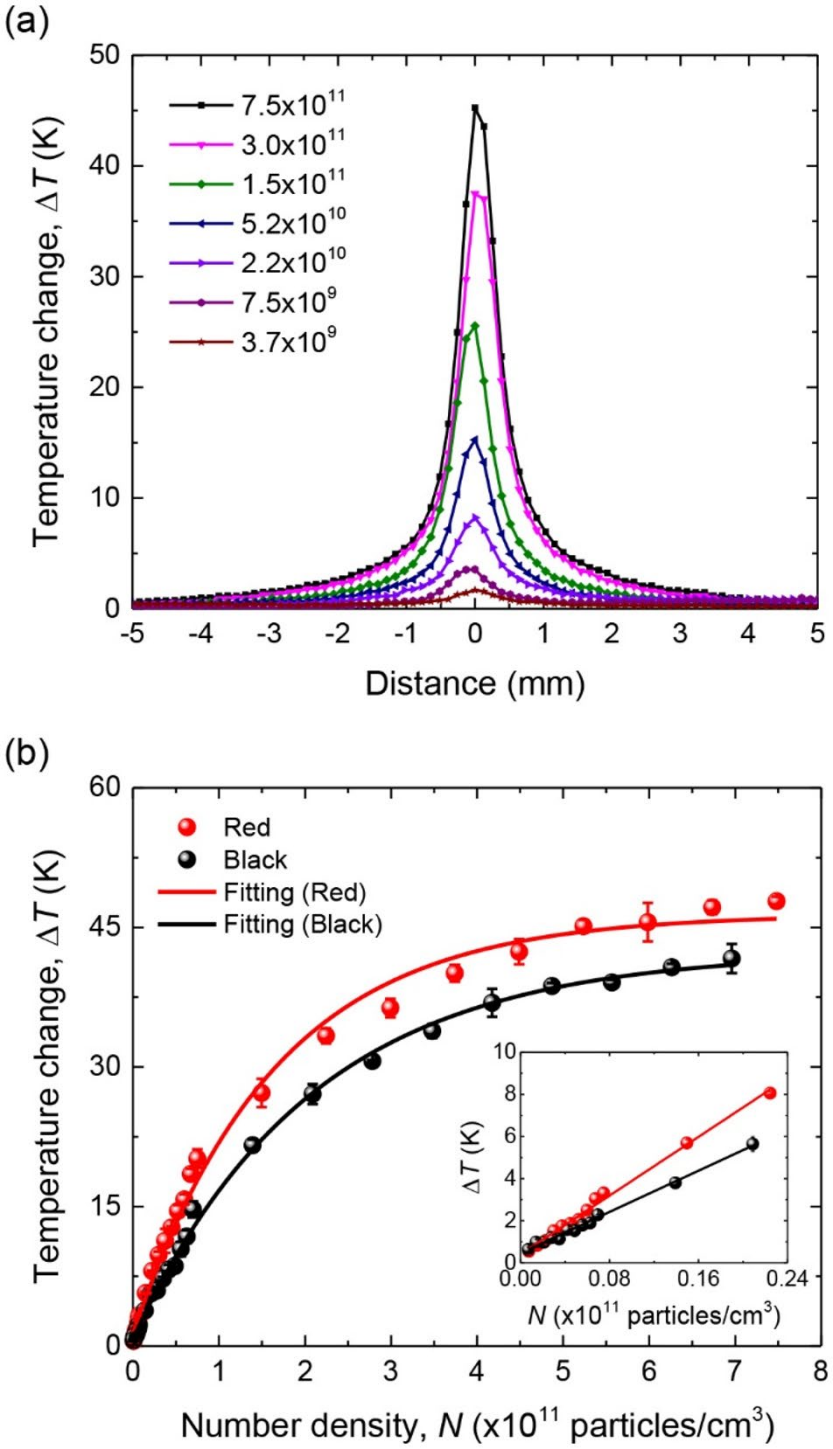
Photothermal detection of CLBs on NC membrane. (**a**) Variations of the spatial temperature profiles of laser-irradiated red latex beads on NC membrane with their number densities. (**b**) Dependence of the peak temperature rises of laser-irradiated red and black latex beads on their number densities. The effective absorption cross sections of both particles are obtained by fitting the experimental data to Eq. (5) in text. Inset: enlarged view of the data in low number density regions. Solid curves are best fits of the experimental data with a linear function. The small $\Delta T$ offset at $N$ = 0 is due to laser heating of blank samples on NC membrane.

Our experiment started with a temperature measurement for a sample spot prepared with concentrated CLB solution (4% w/w) after serial dilution and excited by a 520-nm laser at a power of 10 mW. Figure 6a shows the spatial temperature profiles of the CLBs deposited on NC membrane at different number densities. The shapes of all profiles are essentially the same, independent of $N$, indicating that the addition of CLBs on the strips does not change much the thermal properties of the systems. What is more noteworthy in the figure is that the temperature rise increased nearly linearly with the CLB number density at small $N$ but was gradually saturated at $N$ = 4 × 10^11^ particles/cm^3^ (Fig. 6b). To interpret this observation, we consider a simple model with the approximation that the magnitude of the temperature change is linearly related to the amount of the energy absorbed by the samples^38^, i.e. $\Delta T\propto {I}_{a}={I}_{0}-{I}_{s}-I$, where ${I}_{a}$ is the intensity of the light absorbed by the CLBs. Accordingly, by referring to Eqs. (1) and (4), the temperature change can be approximated by

5 $$\Delta T\approx {CI}_{a}=C\left({I}_{0}-{I}_{s}-I\right)=C{I}_{0}\mathrm{exp}\left(-\tau d\right)\left[1-\mathrm{exp}\left(-{\sigma }_{eff}Nd\right)\right],$$

where the parameter $C$ is a function of laser power, the thermal properties of the substrate, sample volume, and the rate of energy transfer from the heated particle to its environment, as well as the emissivity of the CLB-deposited NC membrane. By fitting the experimental data in Fig. 6b to Eq. (5), we obtained ${\sigma }_{eff}$ = 6.1 ± 0.3 × 10^–10^ cm^2^/particle for the red CLBs and ${\sigma }_{eff}$ = 4.8 ± 0.2 × 10^–10^ cm^2^/particle for the black CLBs at 520 nm.

The values of ${\sigma }_{eff}$ determined above are about 1.3-fold and 3.2-fold as large as those of red and black beads, respectively, dispersed in the 90% glycerol–water mixture. The difference in the enhancement factor (${\sigma }_{eff}/\sigma$) between these two types of beads is so significant that it is a subject worthy of discussion. In a study for the light absorption properties of dyes in sunscreens, Herzog and Sengün^26^ found that the presence of non-absorbing scattering particles such as sub-micron methylmethacrylate (PMMA) spheres in the samples can cause an increase of the dye absorbance (e.g. from ${A}_{0}$ to ${A}_{\mathrm{p}}$) due to multiple light scattering. This enhancement factor (${{A}_{\mathrm{p}}/A}_{0}$), however, decreases non-linearly with increasing $\sqrt{{A}_{0}}$. The authors attributed the effect to the damping of photon diffusion in the highly scattering medium when the light is absorbed by the dye molecules. The stronger the absorption is, the faster is the damping and therefore the smaller is the enhancement factor. In the present experiments, since ${A}_{0}$ is linearly proportional to $\sigma$ and ${{A}_{\mathrm{p}}/A}_{0}$ is linearly proportional to ${\sigma }_{eff}/\sigma$ for samples prepared under the same conditions, our observation that the red beads have a larger $\sigma$ than the black beads but a smaller ${\sigma }_{eff}/\sigma$ than the black beads at 520 nm is consistent with their findings. Thanks to the significant enhancement of the absorption, we were able to detect readily the thermal signals of the black latex beads on NC membrane, despite that the absorption band of these CLBs peaks at ~ 620 nm, which is deviated from the excitation laser wavelength of 520 nm by about 100 nm (Fig. 5).

Although the detection of CLBs on NC membrane by PTD is significantly enhanced by the multiscattering effect, the trade-off is that its effective excitation laser intensity is considerably reduced. Following Eq. (5), we write the heat generation term commonly used in Fourier’s law of thermal conduction as

6 $$Q=\frac{{I}_{a}}{d}=\frac{{I}_{0}\mathrm{exp}\left(-\tau d\right)}{d}\left[1-\mathrm{exp}\left(-{\sigma }_{eff}Nd\right)\right]=\frac{{I}_{eff}}{d}\left[1-\mathrm{exp}\left(-{\sigma }_{eff}Nd\right)\right],$$

where $Q$ is the amount of heat generated (W/m^3^) and ${I}_{eff}\equiv {I}_{0}\mathrm{exp}\left(-\tau d\right)$ is the effective laser intensity (W/m^2^). Given a laser power of 10 mW and $\mathrm{exp}\left(-\tau d\right)$ = 0.20, we calculated the laser intensities to be ${I}_{0}$ = 10 W/cm^2^ and ${I}_{eff}$ = 2.0 W/cm^2^, which suggests an attenuation of the incident laser power by 80% due to the strong light scattering by the membrane. Interestingly, the loss is compensated by a gain (${\sigma }_{eff}/\sigma$) of the optical path length of the excitation laser light in the medium. Further increase of the gain is possible since an enhancement of the path length by a factor of 7 has been previously reported for dye solution added with polystyrene beads^27^.

The ability to perform quantitative analysis is an essential feature of TLFIA. We demonstrated the ability by fitting the experimental data with a linear line over the low number density region of CLBs (inset in Fig. 6b). The good fit indicates that the technique is suitable for quantitative purpose. For experiments conducted with a laser power of 10 mW, we obtained a slope of 3.8 × 10^–10^ K cm^3^/particle and 2.7 × 10^–10^ K cm^3^/particle for red and black latex beads, respectively. Based on the fact that the highest temperature resolution of the infrared camera is 0.1 °C, it suggests an ultimate sensitivity of about 3 × 10^4^ particles/mm^2^ for both particles on the 0.1-mm-thick NC membrane. This sensitivity is comparable to that of fluorescent nanodiamonds (diameter of 100 nm) reported in our previous work using a magnetic modulation technique to achieve background-free detection^39^. However, in practice, we found that the sensitivity is limited by the fluctuations (~ 1 °C over 10 min) of room temperature in our lab^40^, which deteriorated the LOD to about 1 × 10^5^ particles/mm^2^ for both CLBs.

### CLB-based COVID-19 testing

**Figure 7:**
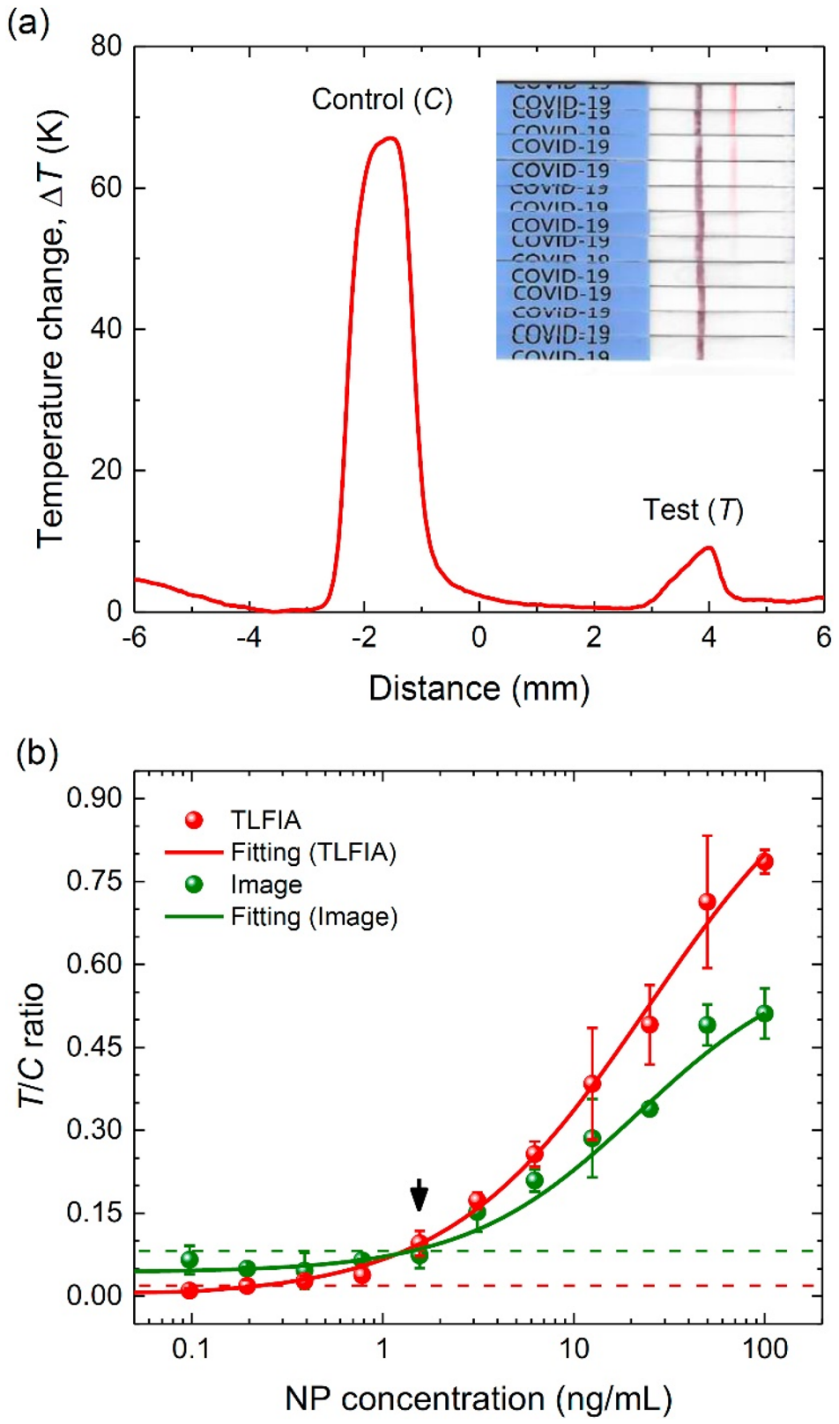
COVID-19 testing with TLFIA. (**a**) Temperature profile of CLBs captured on a COVID-19 antigen test strip, obtained with the TLFIA reader. The assay is conducted for NPs of the SARS-CoV-2 virus at the concentration of 1.56 ng/mL. Integrated areas of the two peaks denoted by “Control (*C*)” and “Test (*T*)” are calculated to obtain the $T/C$ ratio. Inset: photograph of COVID-19 antigen test strips after the assays over the NP concentration range of 100–0 ng/mL (top to bottom) through twofold serial dilution. (**b**) Comparative LFIA for SARS-CoV-2 NPs by thermometric detection, image analysis, and color visualization with the rapid antigen test strips for COVID-19. Solid curves are best fits of the experimental data to logistic functions. The black arrow indicates the LOD of visual inspection and the green and red dashed lines indicate the LODs of image analysis and TLFIA, respectively.

After laying a solid foundation for the TLFIA platform, we applied it for quantitative diagnostics of the nucleocapsid proteins (NPs) of SARS-CoV-2 using commercially available rapid antigen test strips for COVID-19. Specifically, the strips employed red latex beads (0.4 μm in diameter) to mark the test lines and black latex beads (0.4 μm in diameter) to mark the control lines. Shown in Fig. 7a is the result of a representative measurement for the spatial temperature profiles of both control and test lines of the COVID-19 antigen test strip after LFIA. The strip was irradiated with a 520-nm laser at a power of 40 mW to enlarge the temperature changes. As expected, both the CLBs could absorb the green light, leading to temperature rises. Comparing these two temperature rises with the data presented in Fig. 6b, we estimated that the amounts of black and red latex beads captured on the control and test lines of the strip were 1.3 × 10^7^ particles/mm^2^ and 8.6 × 10^5^ particles/mm^2^, respectively, for the assay conducted at the NP concentration of 1.56 ng/mL.

In the inset of Fig. 7a, we also show a photograph of the strips after the assays over a wide NP concentration range of 0–100 ng/mL. The LOD of color visualization with the naked eye was about 1.5 ng/mL. To carry out quantitative analysis of the TLFIA data, we obtained the integrated areas of the two peaks, as denoted by “Control (*C*)” and “Test (*T*)” in Fig. 7a, and took their ratios for all the temperature profiles. This allowed us to reduce systematic errors appearing during the measurements. The LOD of the assays was estimated from the 3 standard deviations of the mean of the blank experiment. With the $T/C$ ratios presented in Fig. 7b, we were able to detect NPs in the sample solution with a concentration as low as ~ 0.1 ng/mL, which is about 10× better than those of visual inspection and conventional image analysis as shown in the same figure^41^. It has been well documented in literature that each SARS-CoV-2 virion contains 35–40 viral RNA–protein (vRNP) complexes and within the individual vRNP, about 800 nt of the genomic RNA are wrapped around 12 copies of NP^42,43^. Given a molecular weight of ~ 100 kDa for the NP dimer^44^, the LOD of ~ 0.1 ng/mL suggests a detection limit of ~ 3 × 10^6^ virions/mL for the SARS-CoV-2 virus.

## Discussion

We have conducted a systematic study for CLBs deposited on NC membrane strips using a home-built TLFIA reader and validated the quantification capability of this photothermal method based on concentration-dependent temperature rises of the beads by laser heating. Our results show a 1.3-fold and 3.2-fold enhancement of the optical absorption at 520 nm by red and black beads, respectively, on NC membrane due to multiple scattering of the light in this highly porous medium. Although the absorption enhancement is accompanied with a fivefold diminution of the incident laser power, the lowest detection limit that we have been able to achieve for both red and black latex beads of ~ 0.4 μm in diameter is 1 × 10^5^ particles/mm^2^. There is still room for further improvement of the detection sensitivity by properly choosing the wavelength of the light source for excitation.

The TLFIA reader developed in this work shares close similarities to that of Bischof et al.^17–19^. However, our instrument is advantageous in having a more compact design, a higher portability, and a lower laser power used. Although the temperature sensing is a highly promising technique, it has several shortcomings. First, the laser power used in TLFIA is high and the safety of the instrument is a concern. Second, the infrared camera is a delicate and costly equipment. Third, the photothermal technique is best used for dry samples. We empirically found that the signal-to-noise ratios of the assays could be increased by a factor of ~ 2 if the strips after use were dried in air for 10 min or more prior to detection (Supplementary Fig. S2). Unfortunately, this sample drying process slows down the speed of the assay, opposite to the purpose of rapid testing. Solving these issues is essential for practical applications of the method in POCT.

This work applies for the first time the concept of temperature sensing to rapid antigen testing for COVID-19. Using nucleocapsid proteins of the SARS-CoV-2 virus as the target antigens, our experiments showed that the CLB-based TLFIA is capable of offering a 10× higher sensitivity than color visualization with the naked eye. It should be emphasized here that the LOD of TLFIA presently determined for the SARS-CoV-2 virus is primarily limited by the large variations between the test strips as well as the non-specific binding among capture and detection antibodies, rather than the intrinsic sensitivity of TLFIA. A significant decrease of the LOD is possible if better manufactured strips and antibody pairs are available. These features, together with further technological improvements, will make TLFIA useful as a rapid and sensitive tool to quantify the levels of SARS-CoV-2 infection and other diseases among patients in clinics and hospitals^45^.

The present study provides a solid basis for the implementation of TLFIA as a quantitative tool for immunodiagnostics with high promises to achieve a detection sensitivity and a measurement accuracy comparable to those of ELISA. It is noted that the theoretical modeling for the multiscattering of light by NC membrane as discussed in this work is general and applicable for fluorescence-based LFIA as well. These assays employ reporters like fluorescent polystyrene beads, quantum dots, and fluorescent nanodiamonds, etc.^39,46,47^, whose detection sensitivity depends on the amount of the light absorbed by the particles, similar to that of PTD. We expect that continuous improvement and process optimization of the technology will open up new avenues and perspectives of LFIA, enabling its practical applications to address a wide range of key issues in life sciences.

## Methods

### Chemicals and materials

Red and black latex beads were from Fisher Scientific, NC membrane was from Millipore, bovine serum albumin (BSA), phosphate-buffered saline (PBS), and all other chemicals were from MilliporeSigma and used without further purification. Both SARS-CoV-2 NPs and COVID-19 antigen test strips were obtained from Panion & BF Biotech, Taiwan.

### Sample preparation

Two types of CLBs were used in this study: red and black latex beads with specified diameters of 0.41 μm and 0.42 μm, respectively. They were deposited on NC membrane strips, each of which was 78 mm long and 4 mm wide with a polystyrene backing of 100 μm in thickness^48^. To prepare the samples for PTD, droplets (0.5 μL/each) of CLB suspensions after serial dilution of the stock solution (4% w/w) were added at the centers of the NC membrane strips to form round spots. The strips were then air-dried and placed in plastic cassettes without covers prior to measurements.

### Instrumentation

The home-built TLFIA reader consisted of a continuous-wave 520 nm laser (OBIS, Coherent) as the excitation source, a sample holder for the plastic cassette, an Arduino UNO board to control a stepper motor mounted on a linear translation stage (SEMC1D-50, SF Technology), and an infrared camera (SC325, FLIR) to detect thermal radiation. A program written in LabVIEW 2020 (Laboratory Virtual Instrumentation Engineering Workbench) scanned the sample across the laser spot and analyzed the data collected by the camera.

### Scanning electron microscopy (SEM)

Structures of the NC membrane (FF120HP Plus, Millipore) were interrogated with a scanning electron microscope (Phenom ProX, Phenom-World) operating at an energy of 15 keV.

### Optical extinction spectroscopy

Extinction spectra of undyed polystyrene microspheres and CLBs in aqueous solution were obtained in 10-mm cuvettes using a standard UV–Vis spectrophotometer (U-3310, Hitachi). Turbidity of the NC membrane was measured in an attenuation mode with the detector positioned at an angle that was 180° relative to the incident light beam. A continuous-wave 520-nm laser (OBIS, Coherent) with an output power of 1 mW served as the light source and a photodiode (SM1PD1B, Thorlabs) with an active area of 10 × 10 mm^2^ behind a Ø9 mm clear aperture detected the signals. Neutral density filters were applied to attenuate the laser intensity to avoid saturation of the detector.

### Immunoassays

The levels of SARS-CoV-2 NPs were measured by using the TLFIA reader, together with commercially available COVID-19 antigen test strips. To carry out the assays, we first prepared the NP solutions in PBS containing ~ 1% BSA after serial dilution with the same buffer, followed by dipping the strips vertically in the sample solutions (100 μL/each) for 15 min to complete the capillary flow. The air-dried strips were finally placed in plastic cassettes for TLFIA measurements. All the experiments were repeated in triplicate.

### Mie scattering calculations

Scattering cross sections of undyed polystyrene microspheres (diameters of 356 nm and 465 nm) dispersed in water were calculated based on the Mie theory. We employed a MATLAB program, similar to that developed by Matzler^49^, and inputted the refractive indexes of polystyrene and water as functions of wavelength ($\lambda$) as^32,50^

7 $${n}_{p}\left(\lambda \right)=1.5663+\frac{0.00785}{{\lambda }^{2}}+\frac{0.000334}{{\lambda }^{4}},$$

8 $${n}_{w}\left(\lambda \right)=1.3231+\frac{0.0033}{{\lambda }^{2}}-\frac{0.000032}{{\lambda }^{4}},$$

where is expressed in microns. The calculated values were then compared with experimental results reported by He et al*.*^32^. After confirming the accuracy of the calculations, the same method was applied to 0.41-µm and 0.42-µm particles in 90% glycerol/water at $\lambda$ = 520 nm only.

### Image analysis

Images of rapid antigen test strips for COVID-19 were acquired with a laser multifunction printer (HP Color LaserJet Pro MFP M479) and color intensities in the green channel were analyzed with the ImageJ software (National Institutes of Health).

## Supplementary Information

Supplementary Figures.

## Data Availability

All the data are available on request from the corresponding author (H.C.C.).
