# Supplementary material for: Thermometric lateral flow immunoassay with colored latex beads as reporters for COVID-19 testing
Source: Sci Rep. 2022 Mar 10;12:3905. doi: 10.1038/s41598-022-07963-1 (PMC8913781; doi:10.1038/s41598-022-07963-1)
Supplement: Supplementary file 1 — Supplementary Figures. [file 41598_2022_7963_MOESM1_ESM.pdf]

Supplementary Information

Thermometric Lateral Flow Immunoassay with Colored Latex Beads as Reporters for COVID-19 Testing

Terumitsu Azuma, Yuen Yung Hui, Oliver Y. Chen, Yuh-Lin Wang, and Huan-Cheng Chang
–

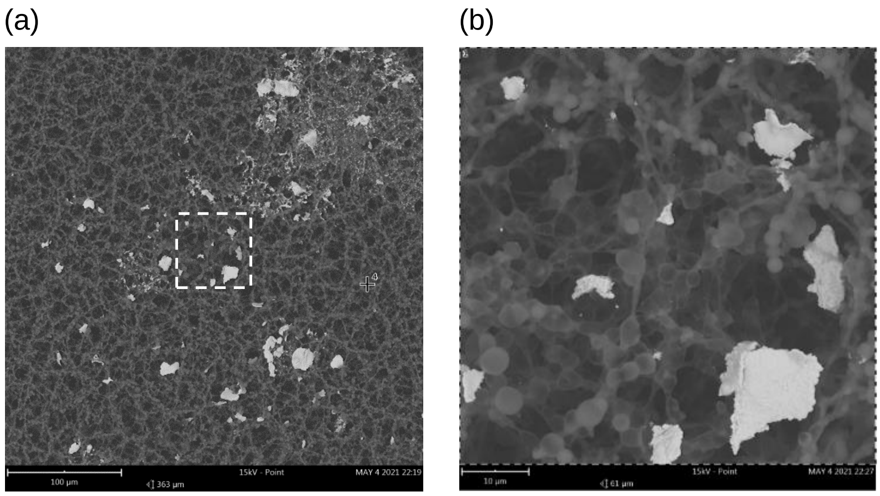

Figure S1. SEM images of 40-nm GNPs deposited on NC membrane with a magnification of 570× (a) and 6600× (b). Scale bars: 100 µm (a) and 10 µm (b). Elemental analysis by energy-dispersive X-ray spectroscopy confirmed that the brighter areas in the dashed box are contributed by GNPs.

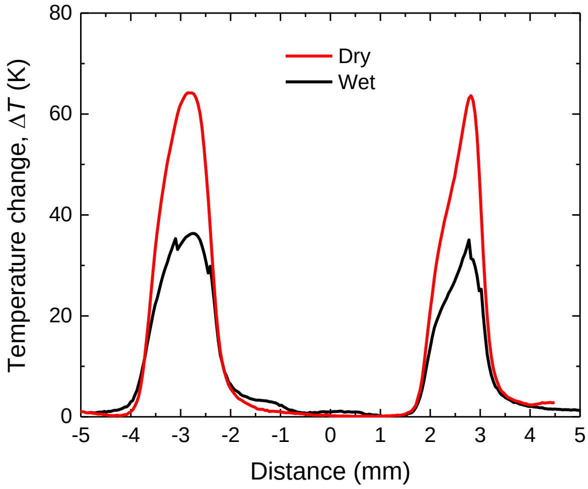

Figure S2. Comparison of the results of rapid antigen testing for COVID-19 using the TLFIA reader for wet and dry strips. The temperatures are measured for the same strip before and after being dried in air. The sample solution contains nucleocapsid proteins of the SARS-CoV-2 virus with a concentration of 100 ng/mL in this experiment.
